# Half of the European fruit fly species barcoded (Diptera, Tephritidae); a feasibility test for molecular identification

**DOI:** 10.3897/zookeys.365.5819

**Published:** 2013-12-30

**Authors:** John Smit, Bastian Reijnen, Frank Stokvis

**Affiliations:** 1 European Invertebrate Survey – the Netherlands, P.O. Box 9517, 2300 RA, Leiden, the Netherlands; 2 Naturalis Biodiversity Centre, P.O. Box 9517, 2300 RA Leiden, the Netherlands

**Keywords:** COI, DNA barcoding, reference database

## Abstract

A feasibility test of molecular identification of European fruit flies (Diptera: Tephritidae) based on COI barcode sequences has been executed. A dataset containing 555 sequences of 135 ingroup species from three subfamilies and 42 genera and one single outgroup species has been analysed. 73.3% of all included species could be identified based on their COI barcode gene, based on similarity and distances. The low success rate is caused by singletons as well as some problematic groups: several species groups within the genus *Terellia* and especially the genus *Urophora*. With slightly more than 100 sequences – almost 20% of the total – this genus alone constitutes the larger part of the failure for molecular identification for this dataset. Deleting the singletons and *Urophora* results in a success-rate of 87.1% of all queries and 93.23% of the not discarded queries as correctly identified. *Urophora* is of special interest due to its economic importance as beneficial species for weed control, therefore it is desirable to have alternative markers for molecular identification.

We demonstrate that the success of DNA barcoding for identification purposes strongly depends on the contents of the database used to BLAST against. Especially the necessity of including multiple specimens per species of geographically distinct populations and different ecologies for the understanding of the intra- versus interspecific variation is demonstrated. Furthermore thresholds and the distinction between true and false positives and negatives should not only be used to increase the reliability of the success of molecular identification but also to point out problematic groups, which should then be flagged in the reference database suggesting alternative methods for identification.

## Introduction

Tephritidae, or true fruit flies, are a large group of flies (Diptera) with some 4 500 species described (Norrbom et al. 1999). The majority of the species are phytophagous. About 35% of them attack soft fruits, including many commercial crops, and some 250 species are considered mild to severe pests (White and Elson-Harris 1992, McPheron and Steck 1996). On the other hand some 40% attack flower heads of or induce galls on Asteraceae, some of which are considered beneficial for the control of invasive weeds outside their natural range (White et al. 1990, White and Elson-Harris 1992, Turner 1996).

Among the economically important taxa five genera have been listed on the quarantine list of the European Union: *Anastrepha* Schiner, 1868, *Bactrocera* Macquart, 1835, *Ceratitis* Macleay, 1829, *Dacus* Fabricius, 1805 and *Rhagoletis* Loew, 1862 (Annex IAI of the Council Directive 2000/29/EC). Most species within these genera are notoriously difficult to identify, therefore the genera are placed on the quarantine list as a whole, despite the fact that not all are pest species. Interceptions on commercial products almost always concern larvae, which are next to impossible to identify. Moreover the number of species that can attack a specific host plant is unknown and the geographic ranges of many species are poorly documented. Therefore there is a desperate need for an alternative method for unambiguous identification of these Tephritid species, especially among plant protection organizations. Hebert et al. (2003) proposed a molecular identification based on a 658 base pair region sequence of the cytochrome *c* oxidase subunit I gene of the mitochondrial DNA (mtDNA), the so-called DNA barcode region (partial COI or *CoxI* gene). Their proposal for the use of the barcoding gene for a molecular identification system initiated the Consortium of the Barcoding of Life (CBOL) in 2004 (http://www.barcoding.si.edu/AboutCBOL.htm). CBOL’s aim is to explore and develop the potential of DNA barcoding for research as a practical tool for species identification. One of the pilot projects was the Tephritid Barcoding Initiative (TBI) with the ambitious aim of gathering barcodes of some 2000 species of fruit flies, focusing mainly on pest and beneficial species. Several studies have been published over the last decade comparing COI sequence datasets with morphological ones for identification purposes among fruit flies, most of which focused on a single genus or a species group within a genus or at most a few closely related genera (Smith-Caldas et al. 2001, Barr et al. 2006, Boykin et al. 2006, Schutze et al. 2007, Nakahara and Muraj 2008, Virgilio et al. 2008, Kohnen et al. 2009, Zhang et al. 2010, Jackson et al. 2011). Virgilio et al. (2012) are the only ones testing DNA barcoding on an extensive dataset of fruit flies, comparable to ours it contains 602 sequences of 153 species. However, it still covers only a limited part of the family, for all species belong to just 10 genera and all are of the same subfamily.

In our study we chose a different approach: instead of focussing on certain species groups or genera, we sequenced as many European species that we could get a hold of, including multiple specimens from distinct geographical populations for as many species as possible. This generated a dataset containing 555 sequences of half of the European species; 124 of the approximately 240 (Smit 2010), from all three subfamilies that are present on the continent. As a result the feasibility of DNA barcoding as an identification tool could be tested over a wide range of species within the family, meanwhile providing a significant contribution to the COI dataset of the Tephritid barcoding database based on morphologically identified specimens. Additional aims were to shed some light on the amount of inter- versus intraspecific variation over a large dataset of fruit fly species belonging to various tribes from different subfamilies as well as testing the phylogenetic signal within the COI barcoding gene.

## Material and methods

### Specimen acquisition

Data on the voucher specimens are provided in Appendix. The vast majority of specimens was collected throughout Europe in 2009 (n = 494). Specimens were directly stored in ethanol 96%. Some of the older material, collected before 2009, has been either directly collected in ethanol 96% (n = 23) or was collected with a Malaise trap (ethanol 70%) and later transferred to ethanol 96% (n = 38).

The oldest material included in this study is from 1999, collected in Kyrgyzstan by Valery Korneyev; this material was stored in 70% ethanol until DNA extraction and amplification. Of the 18 specimens collected, only four resulted in full barcode sequences, hence these are the only ones included in the dataset.

We have included up to eight specimens from geographically distinct populations in order to test the intraspecific variation for as many species as possible. However, we were unable to obtain more than one specimen for a number of species, whereas we have included between 9 and 15 specimens for species with uncertain taxonomy due to species complexes or host races (Table 1). For *Chaetostomella cylindrica* (Robineau-Desvoidy, 1830) we included 23 specimens in order to cover as much of the host races as possible (Knio et al. 2007, Smith et al. 2009).

**Table 1.** The number of species with their range of specimens included in our dataset.

Specimens per species	No. species
1	41
2–8	78
9–15	15
> 15	1

The dataset contains 13 specimens of 11 species originating from Peru, some of which have their congeners among European taxa. These were added to see whether these more distant related taxa have any affect the molecular identification of a dataset of primarily European species. Thus adding a second geographical scale, besides multiple populations per species.

Additionally one outgroup specimen from the closely related family Ulidiidae was used to root the tree: *Ulidia nigripennis* Loew, 1845.

The dataset includes 554 sequences of 135 ingroup species from three different subfamilies and 42 genera and one outgroup sequence.

### DNA extraction and amplification

**Figure 1.**
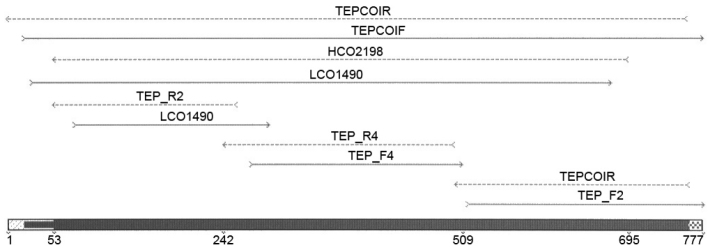
Primer positions within the COI region.

One or two legs per specimen were used for genomic DNA extraction using the 96 wells Qiagen DNeasy Blood and Tissue Kit with a modified protocol. Due to the small size of the legs the tissue was manually ground with a disposable pestle in a 1.5 ml tube. The lysate was transferred to 96 well plates. Elution was performed in 50 µl elution buffer. 658 bp products were amplified using PCR primers LCO1490 and HCO2198 (Folmer et al. 1994) in most specimens. Amplification failed in some specimens therefore different primer sets were developed based on the full mitochondrial genomes of *Bactrocera oleae* (Rossi, 1790) (GU108464) and *Ceratitis capitata* (Wiedemann, 1824) (AJ242872) obtained from GenBank. Primers can be found in Table 2, their corresponding positions within the COI region are depicted in Figure 1.

**Table 2.** Primer pairs used for amplification of the COI marker.

Primer name	Primer sequence	Length (in bp)
L1490 (Folmer et al. 1994)	5’ - GGTCAACAAATCATAAAGATATTGG - 3’	658
H2198 (Folmer et al. 1994)	5’ - TAAACTTCAGGGTGACCAAAAAATCA - 3’	
TEP_F2	5’ - TAGGAGCAGTAAATTTTAT - 3’	(+H2198) 211
TEP_R2	5’ - CAAAAACTTATATTATTTAT - 3’	(+L1490) 241
TEP_F4	5’ - ATTATAATTGGAGGATTTGG - 3’	268
TEP_R4	5’ - GTAATTCCTGTTGATCGTATATTAAT - 3’	
TEPCOIF	5’ - TAAACTTCAGCCATTTAATC - 3’	777
TEPCOIR	5’ - TTTTCCTGATTCTTGTCTAA - 3’	

The 25 μl PCR reaction mixes contained 18.75 μl of ddH_2_O, 2.5 μl of 10 × CoralLoad PCR Buffer (Qiagen), 1 μl of each primer (10 pM), 1.25 U of Taq DNA Polymerase (Qiagen), 0.5 μl of dNTP’s and 1 μl of DNA template. The amplification protocol consisted of 3 min at 94 °C followed by 40 to 50 cycles of 15 s at 94 °C,30 s at 60 °C to 35 °C and 40 s at, 72 °C and a final 5 min at 72 °C.

Direct sequencing was performed at Macrogen, Korea on a ABI 3730XL sequencer.

### Data analysis

Sequences recovered did not contain any insertions, deletions, or stop codons. 555 specimens representing 136 different species from various geographical locations were included in the dataset, resulting in a final alignment of 554 ingroup taxa and a single outgroup. Sequences were assembled and adjusted with Sequencher v4.10.1 (Gene Codes Corp.). Bioedit version v7.0.9.0 (Hall 1999) was used to align the sequences and MacClade version v4.08 (Maddison and Maddison 2000) was used to check for stopcodons. All sequence data, additional geographic and ecological data as well as photographs of the specimens were uploaded to the BOLD database, which ID codes are included in Appendix.

### Molecular identification

The Neighbour-Joining analyses were performed using MEGA5 (Tamura et al. 2011). Distance analysis was conducted using the Kimura 2-parameter model (K2P) (Kimura 1980), and will simply be referred to as distance. The values given in brackets after the mean distance are ranges. The number of informative nucleotide characters in the dataset was 302. Success of the NJ tree-based identification (NJT) is assessed as described Hebert et al. (2003); i.e., sequences were considered successfully identified as long as they formed species-specific clusters. Species with sequences at multiple positions in the tree were considered misidentifications and singletons were counted as ambiguous. Second we used the revised criteria (NJT_M) as described by Meier et al. (2006); where identification is considered successful when a sequence is found at least one node into a cluster of exclusively conspecific sequences or in a polytomy with conspecifics. Species with sequences at least one node into an allospecific cluster or polytomy of allospecific sequences are considered misidentifications. Singletons, sequences as a sister group to conspecifics as well as sequences within a polytomy with at least one conspecific and allospecific sequence are considered ambiguous.

Additional to the tree-based identification we used an identification based on direct sequence comparison by using each sequence as a query to all other sequences in the dataset. SpeciesIdentifier v1.7.8 (Meier et al. 2006) was used to calculate distances, to find the closest barcode match and to determine the threshold value below which 95% of all intraspecific distances are found. The identification criteria used are ‘Best Match’ (BM) and ‘Best Close Match’ (BCM) as described by Meier et al. (2006). The identification is considered successful in BM when the closest match is from the same species. When the species are different it is considered a misidentification. Several equally good best matches from more than one species is considered ambiguous. In BCM the criteria are the same as BM, but the results have to fall within the 95^th^ percentile of all intraspecific distances.

Finally we included the “All species barcodes” (ASB) criteria as described by Meier et al. (2006). This analyses uses the same threshold as used in BCM and identifications were only considered successful when all conspecific sequences top the list of best matches. When at least one allospecific sequence is more similar than the least similar conspecific sequence identification is considered ambiguous, if the query is more similar to all sequences from another species it is considered a misidentification.

Virgilio et al. (2012) introduced a method to improve the accuracy of the interpretation of the success-rates by distinguishing between true and false positives and negatives. True positives (TP) are the queries that have been correctly identified and are below the threshold value, false positives (FP) are incorrectly identified and below the threshold value. True negatives (TN) are correctly rejected because they are misidentified and above the threshold value, false negatives (FN) are correctly identified queries that are rejected because their distance is above the threshold value. Distinguishing these categories allows statements on the accuracy ((TP+TN/n.queries), precision (TP/(TP+FP)), overall ID error ((FP+FN)/n.queries) and relative ID error (FP/(TP+FP)), see Virgilio et al. (2012). These values are assessed for the dataset at hand.

## Results

### DNA extraction and amplification

The DNA of the majority of the specimens could be amplified with the standard PCR primers (Folmer et al. 1994). However, 23 out of the 555 samples needed alternative primers (Table 2). Nearly half only needed one alternative primer (Table 3), whereas others, like the Kyrgyzstan material, needed a cocktail of primers and the amplification protocol needed adjustment as given above.

**Table 3.** The species for which alternative primers have been used for DNA amplification.

Taxon (no specimens)	Probable reason for failure	Used primer(s)	Additional sequences with Folmer et al. (1994)
*Acanthiophilus walkeri* (1)	DNA degraded, specimen stored in ethanol 70% for 7 years	All	0
*Bactrocera oleae* (1)	DNA degraded, specimen stored in ethanol 70%	All	1
*Plaumannimyia* sp. (1)	?	TEPCOI	0
*Rhagoletis cerasi* (1)	?	TEPCOI	4
*Rhagoletis cingulata* (3)	Taxon-specific mutation at primer site?	TEPCOI	0
*Rhagoletis samojlovitshae* (1)	DNA degraded, specimen stored in ethanol 70% for 10 years	All	0
*Sphenella marginata* (7)	Taxon-specific mutation at primer site?	TEPCOI, TEP_F2, TEP_R2 & Folmer et al. (1994)	0
*Tephritis nebulosa* (1)	DNA degraded, specimen stored in ethanol 70% for 10 years	All	0
*Terellia colon* (1)	?	TEPCOI	11
*Terellia luteola* (1)	DNA degraded, specimen stored in ethanol 70% for 10 years	TEPCOI, TEP_F2, TEP_R2 & Folmer et al. (1994)	1
*Trupanea* cf. *metoeca* (1)	DNA degraded, specimen stored in ethanol 70% for 2 years	TEPCOI	0
*Trypeta artemisiae* (2)	?	TEPCOI	1
*Ulidia nigripennis* (1)	?	TEPCOI	0
*Urophora ivannikovi* (1)	DNA degraded, specimen stored in ethanol 70% for 10 years	All	0

### Sequence alignment and analyses

**Figure 2.**
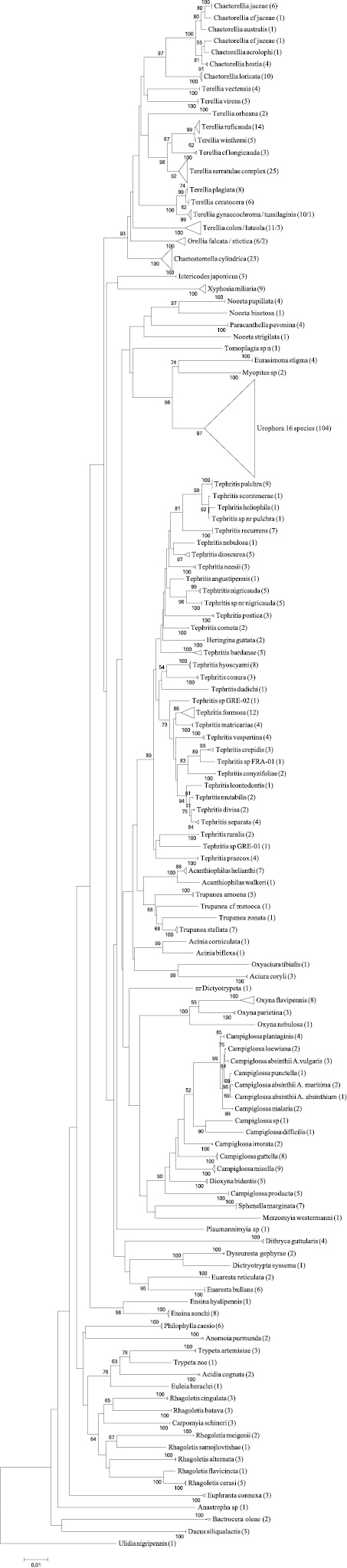
The Neighbour-Joining tree of the entire dataset based on COI barcodes. Terminal branches have been collapsed in order to save space, the total number of specimens is given in brackets and the area surface of the triangle represents the amount of variation. When a terminal branch contains two species, both names are provided as well as their respective number of specimens. If a branch contains more than two species only the number of species as well as the number of specimens are given. Bootstrap values above 50 (1000 replicates) are given at the nodes.

The data are presented in a Neighbour-Joining tree only (Figure 2) for we are merely interested in a distance-based clustering of species based on similarity of the sequences and not a character based clustering of the sequences. Despite the fact that the NJ tree fits very well to both the morphological phylogenetic tree (Korneyev 2000) as well as the recent molecular ones (Han et al. 2006, Han and Ro 2009) it is stressed here that this tree may not reflect the true phylogenetic tree, because running the data through a Maximum Parsimony (MP) and Maximum Likelihood (ML) analyses result in different topologies.

We only focus on the feasibility of DNA barcoding for molecular identification, any probable taxonomic implications of the data generated are not dealt with in this paper.

### Molecular identification

**Figure 3.**
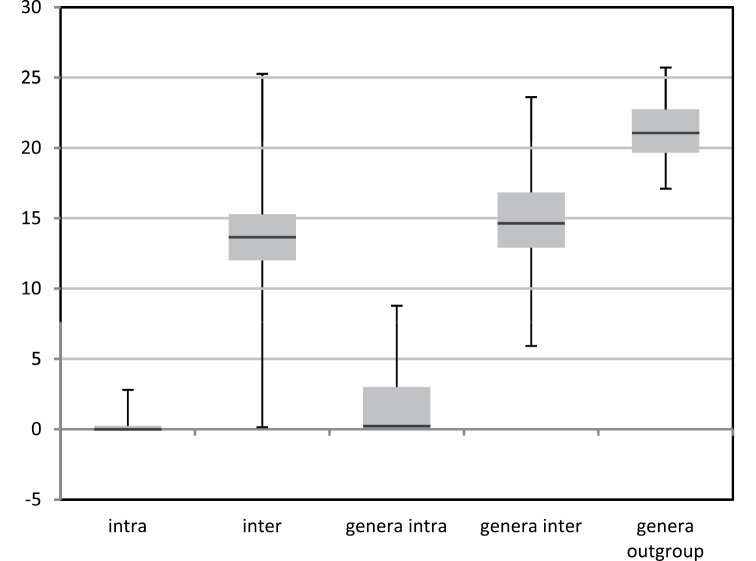
Box plots depicting the variation in mean distances using K2P-distance modeling of sequence divergence for intraspecific, interspecific difference among the species and genera, as well as the ingroup genera with the outgroup genus.

With some exceptions the COI barcodes in general seem to provide a good molecular marker for identification of European fruit fly species. The mean distances between species was on average 13.2% (0.15–25.27%) whereas within a species this was a mere 0.24% (0–2.80%) (Figure 3). There is no clear barcode-gap for 2.7% of all pairwise comparisons fell between the minimum interspecific distance (0.15%) and the maximum intraspecific distance (2.8%). Among the genera the mean distances were 1.49% (0–8.78%) within and 14.96% (5.92–23.61%) between the genera. The distances between the ingroup genera and the outgroup was 21.18% (17.11–25.72%).

Identification success-rates of all five criteria are given in Table 4. Several species groups within the genus *Terellia* Robineau-Desvoidy, 1830 and apparently none of the species of *Urophora* Robineau-Desvoidy, 1830 could reliably be identified using COI barcodes.

**Table 4.** Identification rates of all five criteria: Neighbour-Joining (NJT) sensu Hebert et al. (2003), revised criteria (NJT_M) according to Meier et al. (2006), and Best Match (BM), Best Close Match (BCM) and All Species Barcodes (ASB) also described by Meier et al. (2006).

Criteria	Correct ID	Ambiguous	Incorrect ID	No match
NJT	63.25%	7.38%	29.37%	-
NJT_M	61.89%	36.22%	1.80%	-
BM	78.19%	12.25%	9.54%	-
BCM (threshold 0.3%)	73.33%	10.45%	3.06%	13.15%
ASB (threshold 0.3%)	59.63%	27.02%	0.18%	13.15%

### Tree-based identification

Both criteria NJT and NJT_M give comparable results with the correct identified sequences: 351 and 344 sequences respectively (Table 4). The main difference is among the number of incorrect and ambiguous sequences, for multiple placement immediately identifies the sequences as incorrect according to NJT, whereas if they still have conspecifics at the different nodes they are regarded as ambiguous according to NJT_M: 41 and 163 versus 201 and 10 sequences.

The Neotropical taxa with European congeners clustered within the appropriate genus, often with a distance greater than those among the European taxa of that particular genus.

*Campiglossa absinthii* (Fabricius, 1805) is placed at three different branches within the NJ tree with slightly lower though similar mean distances as among the other closely related species (Table 5). All three groups originate from different *Artemisia* host-plants and might therefore represent different host-races, or perhaps even different species. Host-plant names are given in Figure 2 and are abbreviated in Figure 8.

Furthermore the NJ analysis places the genus *Dioxyna* Frey, 1945 within the genus *Campiglossa* Randani, 1876 and *Heringina* Aczél, 1940 within *Tephritis* Latreille, 1804 both of which are corroborated with the ML and MP analyses.

**Table 5.** Mean K2P-distances in percentages between the species of the *Campiglossa loewiana*-group.

*Campiglossa malaris*						
*Campiglossa absinthii* / on *A. vulgaris*	1.07					
*Campiglossa loewiana*	1.23	0.46				
*Campiglossa punctella*	1.23	0.77	0.92			
*Campiglossa absinthii* / on *A. absinthium*	1.23	0.77	0.92	0.30		
*Campiglossa absinthii* / on *A. maritima*	1.38	0.92	1.08	0.46	0.46	
*Campiglossa plantaginis*	1.54	0.76	0.61	0.92	0.92	1.07

### Similarity-based identification

Under the BM criteria 434 sequences were regarded as correctly identified, 53 incorrectly and 68 as ambiguous. The dataset contains 394 sequences with a closest match at 0%, 56 (14,21%) of them having an allospecific identical match.

The threshold for the 95^th^ percentile of the intraspecific distances has been calculated at 0.3%. Success under BCM is 73.33% (84.44% of the non-discarded queries), whereas 17 sequences were regarded as incorrectly identified, 58 ambiguous and 73 did not have a match below the threshold, the proportions of TP, FP, FN and TN were 0.733, 0.135, 0.048 and 0.082 respectively.

Under the ASB criteria 331 sequences were correctly identified, 150 were ambiguous, one was misidentified and, like BCM, 73 did not have a match below the threshold.

## Discussion

### Molecular identification

**Figure 4.**
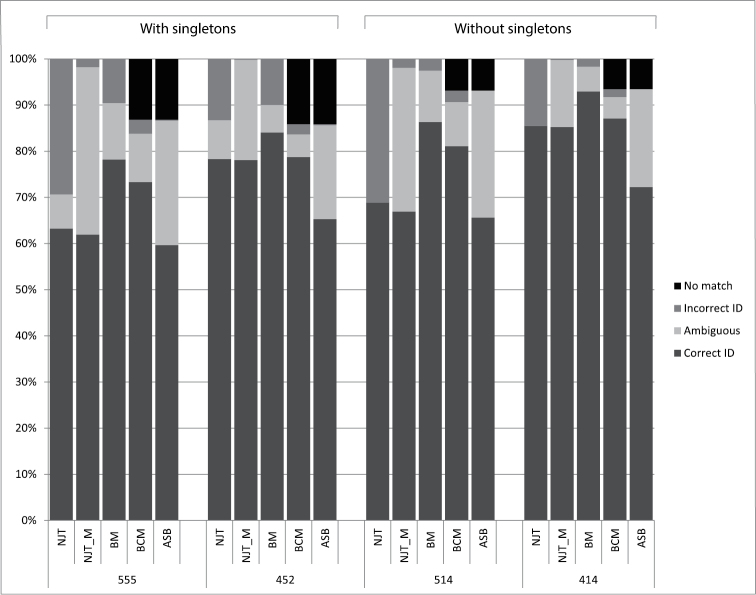
Identification rates of all five criteria: Neighbour-Joining (NJT) sensu Hebert et al. (2003), revised criteria (NJT_M) according to Meier et al. (2006), and Best Match (BM), Best Close Match (BCM) and All Species Barcodes (ASB) also described by Meier et al. (2006) for four different datasets, including singletons and with (n = 555) or without (n = 452) *Urophora*, and the same excluding singletons (n = 514) and (n = 414) respectively.

The discussion is confined to the success-rates of the tree-based identification criteria NJT_M and the similarity-based identification according to the BCM criteria. The numbers are given for the other criteria as well but they are not discussed further (Figure 4). The NJT criteria gives an overrepresentation of incorrectly identified sequences, whereas BM seems to have an overoptimistic prediction of correctly identified sequences (Figure 4) (Meier et al. 2006, Virgilio et al. 2012). Like BM the ASB criteria does not take into account the possibility of multiple haplotypes for a single species and regards them, contrary to BM, as ambiguous instead of incorrect identified (Figure 4) (Meier et al. 2006).

The low success-rate is in part due to singletons and the genus *Urophora*. Of the 135 species 38 (41 when three *Urophora* singletons are included) cannot have a match simply because they lack conspecifics (7.39% of the sequences) (Meier et al. 2006, Virgilio et al. 2010, 2012). Deleting them from the dataset as to simulate a perfect world scenario with 100% taxon-coverage, for every sequences has at least one conspecific, results in a higher success-rate, increasing 5.03% and 7.72% respectively and nearly halves the discarded queries (Figure 4). *Urophora* makes up 18.56% of the entire dataset. Deleting them results in different identification-rates, for which success increases a staggering 16.21% in NJT_M and 5.43% in BCM (Figure 4). Combining the two, e.g. deleting both the singletons and *Urophora*, provides an increase correct identified queries of 23.38% and 13.77% respectively (Figure 4). Comparing these identification-rates it becomes clear that *Urophora* is largely responsible for the lack of success with molecular identification in this dataset. The ambiguity caused by the *Urophora* sequences here is due to the fact that there are not only conspecific sequences per species but also in several cases per population. These of course are identical but in most cases different from conspecific sequences from other populations, interpreted by BCM as ambiguous for they might represent different haplotypes of the same species or are in fact two different species, whereas morphologically they clearly belong to the same species. Moreover more than half of the allospecific matches are caused by the genus *Urophora*, the rest being caused by the problematic *Terellia* groups.

This stripped dataset, e.g. without singletons and without the genus *Urophora*, results in 87.1% of all queries and 93.23% of the not discarded queries as correctly identified, which is similar though slightly lower than the dataset of interceptions of Virgilio et al. (2012).

**Figure 5.**
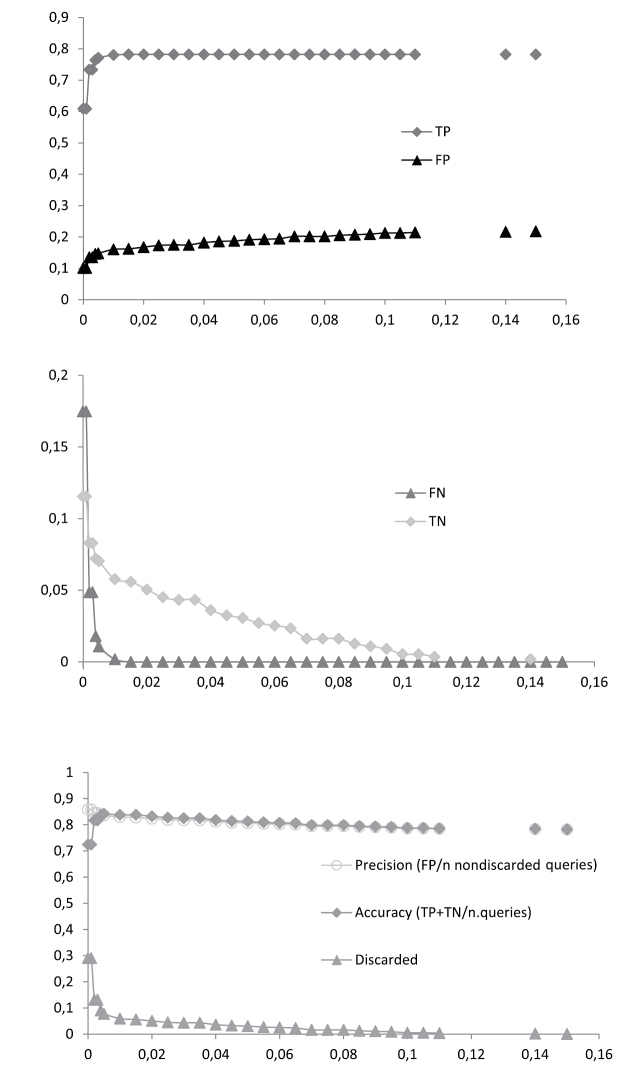
Best Close Match (BCM) identification of the entire dataset (n = 555). Proportions of true positives (TP), false positives (FP), false negatives (FN) and true negatives (TN) are given for 30 arbitrary distance thresholds ranging from 0.15 to 0.00. For each threshold the percentages of precision, accuracy and discarded queries were calculated.

**Figure 6.**
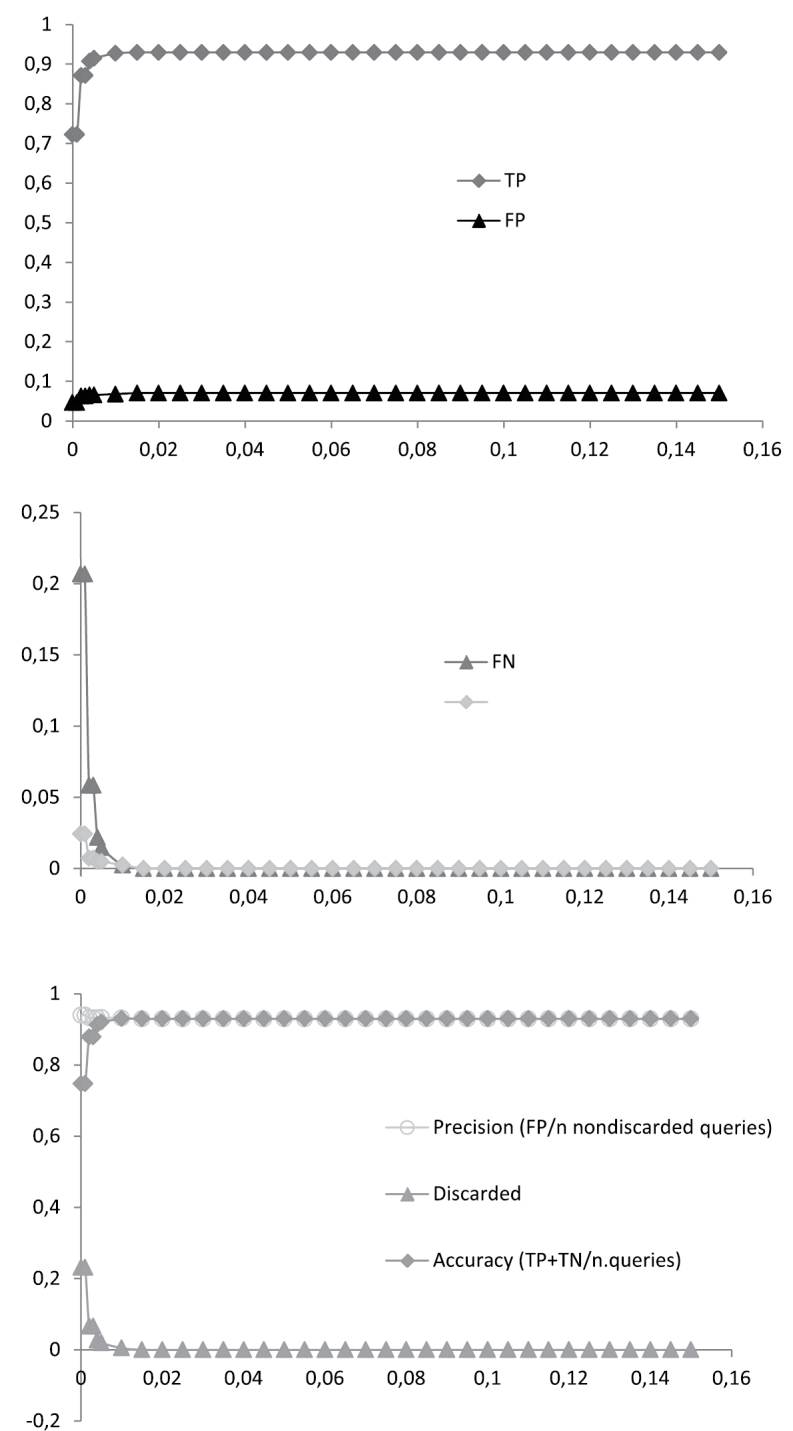
Best Close Match (BCM) identification of the stripped dataset, e.g. excluding singletons and *Urophora* (n = 414). Proportions of true positives (TP), false positives (FP), false negatives (FN) and true negatives (TN) are given for 30 arbitrary distance thresholds ranging from 0.15 to 0.00. For each threshold the percentages of precision, accuracy and discarded queries were calculated.

**Figure 7.**
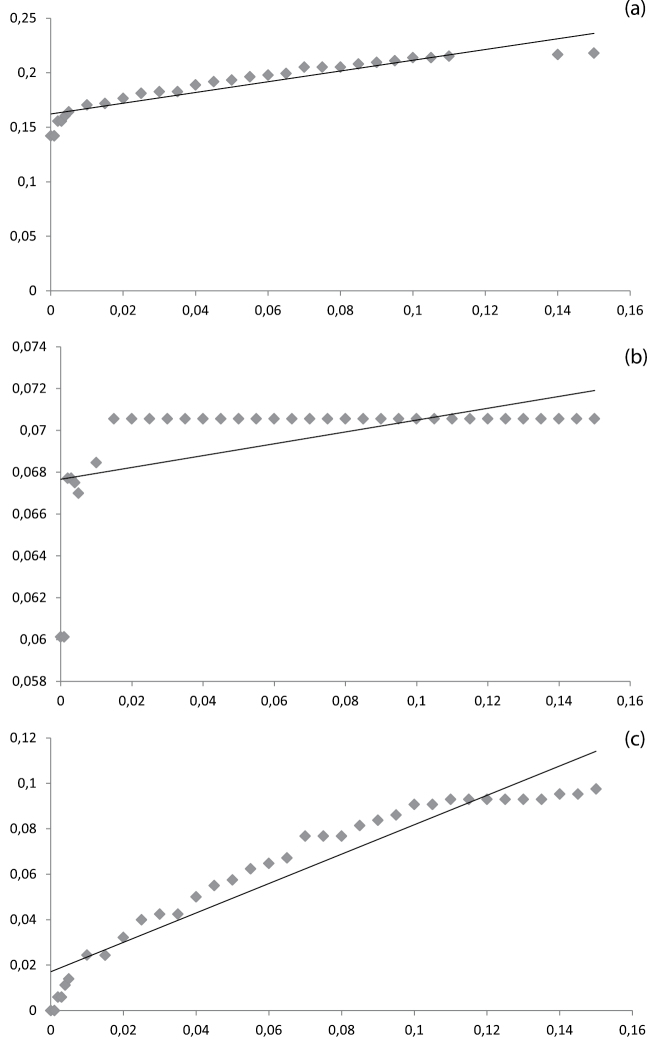
Relative ID errors at 30 arbitrary threshold values for a. the entire dataset (n = 555), b. the stripped dataset, e.g. excluding singletons and *Urophora* (n = 414) and c. the stripped dataset excluding the problematic *Terellia* groups. Linear regression was used to infer the *ad hoc* threshold for the 95^th^ percentile of the correctly identified queries and the relative ID error does not exceed 5%. In (a) and (b) this value is below 0.00, only in (c) this value is positive: 0.051 (R-square 0.91).

The threshold value in BCM is of strong influence on the results, as already noted by Virgilio et al. (2012). The success-rates have been calculated for a range of arbitrary threshold values between the largest observed distance and 0.00 (Figure 5). A rapid increase of accuracy can be seen to 0.84 at a threshold of 0.5%, after which it declines again to 0.78, similarly TP increases and FN decreases. Precision however never exceeds 0.86. Thus when calculating the relative ID error, linear regression shows that for a relative ID error < 0.05 the threshold value is lower than 0.00 (Figure 7a). Even when the stripped dataset is used precision only reaches 0.94 (Figure 6), therefore again producing a threshold value lower than 0.00 for a relative ID error < 0.05 (Figure 7b). This linear regression function is used by Virgilio et al. (2012) to infer the *ad hoc* threshold for the 95^th^ percentile of the correctly identified queries and where the relative ID error does not exceed 5%. When this threshold value is lower than 0.00 the dataset should be regarded as unreliable (Virgilio et al. 2012). Only when the problematic *Terellia* groups are deleted from our already stripped dataset an *ad hoc* threshold value > 0 can be inferred (Figure 7c). Therefore the dataset created here is unreliable for molecular identification. This was also clear by the number of allospecific matches as well as the ambiguity among the success-rates, resulting in an low overall success-rate. Several other groups have recently been studied in which DNA barcoding was shown to have a limited performance (Armstrong and Ball 2005, Kaila and Stahls 2006, Meier et al. 2006, Elias et al. 2007, Neigel et al. 2007, Skevington et al. 2007, Virgilio et al. 2008, Dasmahapatra et al. 2009, Jackson et al. 2011, Barr et al. 2012).

Distinguishing between true and false positives and negatives is based on morphological identification of the voucher specimens. Therefore taxonomic specialists are needed to build and check the reference database that can be used for molecular identification. Adding more morphologically correctly identified specimens will increase the understanding of the limitations of molecular identification for that particular group (Meyer and Paulay 2005, Ekrem et al. 2007, Kwong et al. 2012). Incorrectly identified sequences will be added to the reference database like BOLD, for it is only human to make errors. Introducing threshold values for molecular identification will point out the obviously incorrectly identified specimens (Meier et al. 2006), but will not help with problematic groups containing for example very low interspecific distances or allospecific matches. Based on our dataset we were able to identify some problematic groups causing limitations for molecular identification of Tephritids illustrated by some examples given below.

### Varying mean distances between different species groups of the same genus

**Figure 8.**
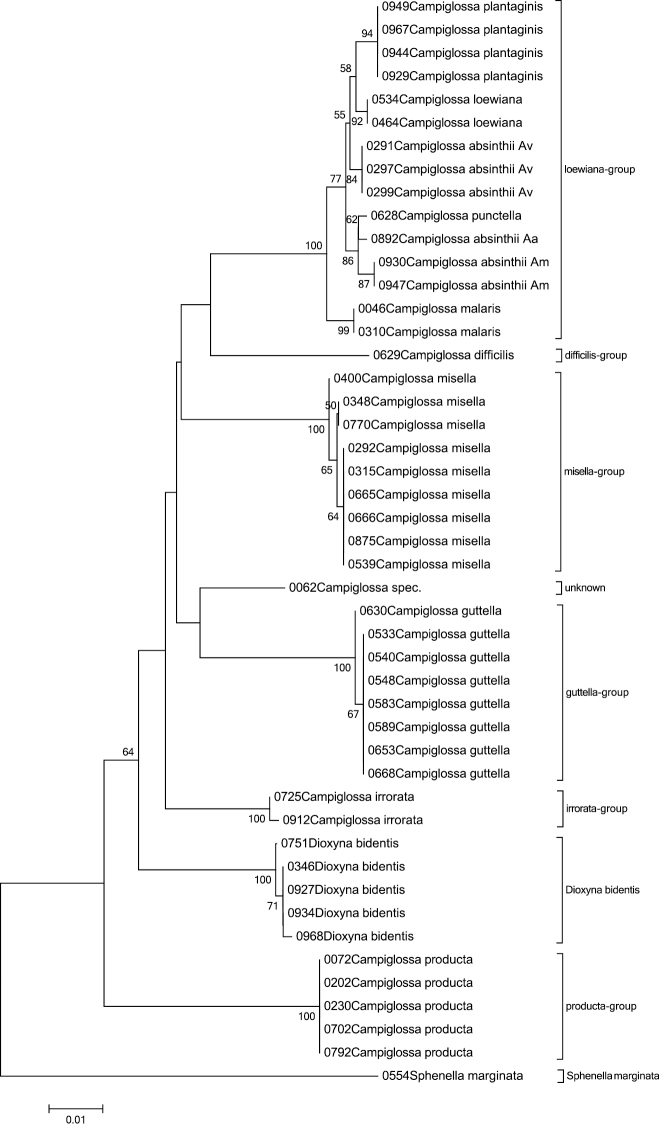
The Neighbour-Joining tree of the genus *Campiglossa* with *Sphenella marginata* as outgroup inferred from COI barcodes. Bootstrap values above 50 (1000 replicates) are given at the nodes.

The species of the genus *Campiglossa* can be identified using DNA barcodes, showing a neat mean distance of 5.2%. Looking in detail, however, shows it has a very broad range of interspecific distances, from 0.3 to 8.7%. Grouping the species into their known morphological species complexes (Merz 1992, 1994) results in a mean distances of 6.2% (4.2–8.6%), because all but one of the groups are represented by just one species (Figure 8). The five species of the *loewiana* group show a mean distance of a mere 0.9% (0.3–1.5%) (Table 5), revealing that these very closely related species are apparently difficult to separate using COI, something which has been noted before in various groups as well as Tephritids (Armstrong and Ball 2005, Kaila and Stahls 2006, Virgilio et al. 2008, Barr et al. 2012, Nieukerken et al. 2012).

Executing a BLAST on the BOLD database with one sequence of *Campiglossa malaris* Séguy, 1938 from our dataset retrieved no less than 18 sequences with a similarity of over 98%, belonging to 5 different species apart from the target species. Excluding *Campiglossa malaris* itself, the sequence with the highest similarity was one belonging to a Nearctic species, *Campiglossa farinata* (Novak, 1974) with a similarity of 99.08%. Furthermore, no less than six sequences showed a similarity of 98.93% belonging to two different species.

These differences in mean distances, especially the short ones among the *loewiana* group, indicate that it is important to include as many sequences of distinct populations per species as possible in a reference database like BOLD to preclude misidentification.

### Multiple specimens

**Figure 9.**
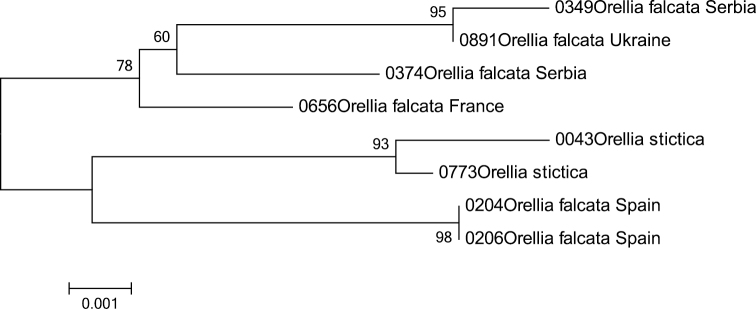
The Neighbour-Joining tree of the genus *Orellia* inferred from COI barcodes. Bootstrap values above 50 (1000 replicates) are given at the nodes.

Adding specimens from geographically distinct populations is necessary in order to shed some light on the intraspecific variation caused by geography (Bergsten et al. 2012). This is clearly illustrated by adding two specimens of *Orellia falcata* (Scopoli, 1763) from Spain, which resulted in a paraphyletic placement, including the second species present in the dataset: *Orellia stictica* (Gmelin, 1790) (Figure 9). Both species are morphologically quite distinct and easy to recognize. Therefore either both species are so closely related that they cannot be separated based on the barcode gene and perhaps a more sensitive marker is needed, or *Orellia falcata* represents a complex of cryptic species.

Likewise it is necessary to add specimens of ecologically distinct populations as well, as is shown by the three ‘host-races’ of *Campiglossa absinthii* and by Smith et al. (2009) for *Chaetostomella cylindrica*.

### Low interspecific variation compared to a high intraspecific variation

**Figure 10.**
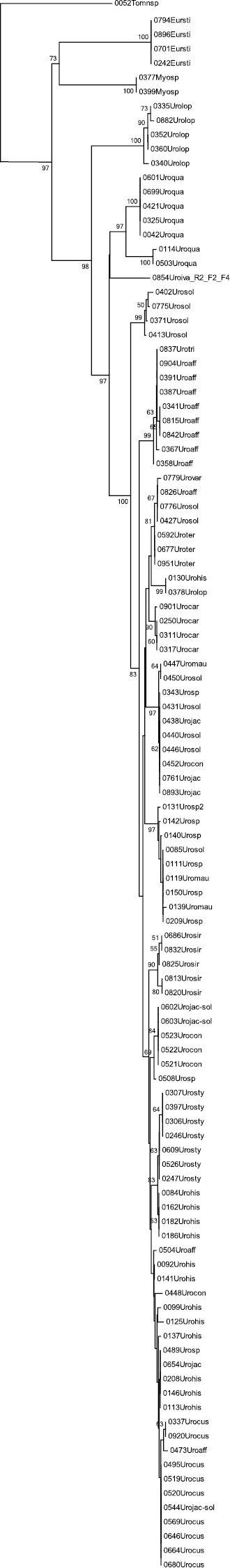
The Neighbour-Joining tree of the genus *Urophora* inferred from COI barcodes. Bootstrap values above 50 (1000 replicates) are given at the nodes.

Looking at the NJ tree (Figure 2, 10) it is immediately obvious that the species of the genus *Urophora* cannot be separated using DNA barcodes. Jackson et al. (2011) already reported that the species of the genus *Urophora* could not be identified using DNA barcodes, having included 10 sequences belonging to three different species. In our dataset we included over 100 sequences of 16 morphologically identified species, resulting in multiple placement of several species and a mean distance of a mere 1.65% (0.3-2.45%). This limited or entire lack of performance of molecular identification is of special interest for it concerns a genus of economic importance with several species regarded as beneficiary for weed control (White and Clement 1987, White and Elson-Harris 1992). Additional genetic markers should be tested for the molecular identification of these species like Elongation Factor 1-α (EF1-α) or ribosomal Internal Transcribed Spacer 2 (ITS2) (Alvarez and Hoy 2003, Farris et al. 2010, Nieukerken et al. 2012).

### The limitations of DNA barcodes for molecular identification

As is shown above, the feasibility of the use of DNA barcodes for molecular identifications relies heavily on the contents of the database used to BLAST against (Meyer and Paulay 2005, Meier et al. 2006, Ekrem et al. 2007, Virgilio et al. 2010, 2012, Kwong et al. 2012). The addition of multiple specimens per species to the database, preferably from geographically distinct populations, as well as different ecologies, provides a much needed insight in the intraspecific versus interspecific variation of the species. Adding more species is a necessity too, because incorporating different species of the *Campiglossa loewiana*-complex clearly demonstrated that the perceived mean distance of 5.2% between the species actually represents the mean distance of the different species groups in this dataset. The mean distance of the species within the *Campiglossa loewiana*-group was a mere 0.9%. Hence threshold values like a ≥ 98% similarity as used by Li et al. (2011) or the 97% used by BOLD for a positive identification do not hold. Introducing the 95^th^ percentile threshold value increases the reliability of the identification success. Further improvement can be achieved by introducing the *ad hoc* threshold as proposed by Virgilio et al. (2012). However, as is shown by our dataset, this is not always possible. Instead of discarding the dataset as unreliable it should be used to identify the problematic groups by looking at the amount of allospecific matches, TP, FP, FN and TN. In that case these problematic groups can be flagged in the reference database so that the user can look for alternative means for identification.

## Conclusion

We conclude that molecular identification of Tephritids using DNA barcoding is possible but should be treated with care due to varying performance within this group as is shown by the dataset analysed here. Even when threshold values are added groups will remain that cannot reliably be identified. We stress that a better performance is strongly dependent on an increasing input of morphologically identified specimens, containing multiple specimens of different geographical populations and different ecologies covering as much of the range of the species as possible, otherwise it remains difficult to detect cryptic species and estimate true diversity. Threshold values for both distance and relative ID error, as well as distinction between positives and negatives, both true and false, should not only be used to improve the reliability of the success for molecular identification but also to identify the problematic groups for molecular identification. These groups should be flagged in the reference database and alternative markers for molecular identification should be tested.

## Appendix

Collection data of all specimens included in this study. (doi: 10.3897/zookeys.365.5819.app) File format: Microsoft Excel file (xls).
